# Supplementary material for: Effects of Post-Ruminal Urea Supplementation during the Seasonal Period on Performance and Rumen Microbiome of Rearing Grazing Nellore Cattle
Source: Animals (Basel). 2022 Dec 8;12(24):3463. doi: 10.3390/ani12243463 (PMC9774649; doi:10.3390/ani12243463)
Supplement: Supplementary file 1 [file animals-12-03463-s001.zip › animals-2027587-supplementary.pdf]

Table S1. Median and IQR of ruminal archaea and bacterial abundance at genera level influenced by the soybean meal (CP) replacement by NPN in the supplement of grazing Nellore steers.

Genera	NNP	IQR	CP	IQR	p-value*
Bacteria;p__Bacteroidota;c__Bacteroidia;o__Bacteroidales;f__Bacteroidetes_BD2-2;g__Bacteroidetes_BD2-2	0.151	0.036	0.112	0.040	0.0027
Bacteria;p__Spirochaetota;c__Spirochaetia;o__Spirochaetales;f__Spirochaetaceae;g__Sediminispirochaeta	0.017	0.011	0.028	0.008	0.00443
Bacteria;p__Firmicutes;c__Clostridia;o__Lachnospirales;f__Lachnospiraceae;g__Lachnospiraceae_ND3007_group	0.554	0.08	0.472	0.120	0.00971
Bacteria;p__Firmicutes;c__Negativicutes;o__Veillonellales-Selenomonadales;f__Selenomonadaceae;g__Schwartzia	0.016	0.007	0.008	0.012	0.01053
Bacteria;p__Bacteroidota;c__Bacteroidia;o__Bacteroidales;f__Rikenellaceae;g__SP3-e08	0.091	0.063	0.143	0.185	0.01508
Bacteria;p__Firmicutes;c__Clostridia;o__Lachnospirales;f__Lachnospiraceae;g__[Ruminococcus]_gauvreauii_group	0.182	0.077	0.297	0.267	0.01737
Bacteria;p__WPS-2;c__WPS-2;o__WPS-2;f__WPS-2;g__WPS-2	0	0	0	0.009	0.02771
Bacteria;p__Firmicutes;c__Clostridia;o__Lachnospirales;f__Lachnospiraceae;g__Lachnospiraceae_NK3A20_group	1.534	0.438	1.746	0.429	0.02987
Bacteria;p__Bacteroidota;c__Bacteroidia;o__Bacteroidales;f__F082;g__F082	3.859	1.349	4.535	1.371	0.02987
Bacteria;p__Firmicutes;c__Clostridia;o__Lachnospirales;f__Lachnospiraceae;g__Lachnoclostridium	0.022	0.018	0.038	0.023	0.04368
Bacteria;p__Proteobacteria;c__Gammaproteobacteria;o__Aeromonadales;f__Succinivibrionaceae;g__Succinivibrionaceae_UCG-002	0.028	0.037	0.02	0.016	0.04369
Bacteria;p__Verrucomicrobiota;c__Lentisphaeria;o__Victivallales;f__vadinBE97;g__vadinBE97	0.014	0.008	0.011	0.006	0.04664
Bacteria;p__Proteobacteria;c__Alphaproteobacteria;o__Rhodospirillales;f__uncultured;g__uncultured	0.028	0.022	0.013	0.019	0.04678
Bacteria;p__Firmicutes;c__Clostridia;o__Lachnospirales;f__Lachnospiraceae;g__Lachnospiraceae	0.208	0.068	0.142	0.079	0.04942
Bacteria;p__Firmicutes;c__Clostridia;o__Lachnospirales;f__Lachnospiraceae;g__probable_genus_10	0.125	0.048	0.098	0.061	0.04942
Bacteria;p__Bacteroidota;c__Bacteroidia;o__Bacteroidales;f__Prevotellaceae;g__Prevotellaceae_UCG-003	1.080	0.418	1.009	0.41	0.05571
Bacteria;p__Firmicutes;c__Clostridia;o__Lachnospirales;f__Lachnospiraceae;g__[Eubacterium]_ruminantium_group	0.752	0.228	0.644	0.236	0.06267
Bacteria;p__Firmicutes;c__Clostridia;o__uncultured;f__uncultured;g__uncultured	0	0	0	0.001	0.06559
Bacteria;p__Actinobacteriota;c__Actinobacteria;o__Bifidobacteriales;f__Bifidobacteriaceae;g__Bifidobacterium	0.012	0.010	0.055	0.093	0.06914
Bacteria;p__Bacteroidota;c__Bacteroidia;o__Bacteroidales;f__PeH15;g__PeH15	0.037	0.034	0.032	0.015	0.07438
Bacteria;p__Firmicutes;c__Clostridia;o__Lachnospirales;f__Lachnospiraceae;g__Stomatobaculum	0.329	0.066	0.280	0.065	0.07868
Bacteria;p__Firmicutes;c__Clostridia;o__Lachnospirales;f__Lachnospiraceae;g__[Eubacterium]_xylanophilum_group	0.148	0.011	0.130	0.042	0.07868
Bacteria;p__Firmicutes;c__Clostridia;o__Peptostreptococcales-Tissierellales;f__Anaerovoracaceae;g__Mogibacterium	0.27	0.084	0.301	0.055	0.07873
Archaea;p__Thermoplasmatota;c__Thermoplasmata;o__Methanomassiliicoccales;f__Methanomethylophilaceae;g__uncultured	0.086	0.113	0.115	0.137	0.07873
Bacteria;p__Firmicutes;c__Bacilli;o__Lactobacillales;f__Lactobacillaceae;g__Lactobacillus	0.013	0.055	0.058	0.114	0.07873
Bacteria;p__Firmicutes;c__Clostridia;o__Lachnospirales;f__Lachnospiraceae;g__Roseburia	0	0.005	0.009	0.023	0.08052
Bacteria;p__Firmicutes;c__Bacilli;__;__;__	0	0.003	0.003	0.007	0.08144
Bacteria;p__Bacteroidota;c__Bacteroidia;o__Bacteroidales;f__Prevotellaceae;g__uncultured	0.143	0.058	0.129	0.053	0.08793
Bacteria;p__Firmicutes;c__Bacilli;o__Erysipelotrichales;f__Erysipelotrichaceae;g__Erysipelothrix	0	0	0	0.002	0.09259
Bacteria;p__Firmicutes;c__Clostridia;o__Lachnospirales;f__Lachnospiraceae;g__[Eubacterium]_cellulosolvens_group	0.033	0.023	0.038	0.028	0.09274
Bacteria;p__Patescibacteria;c__Gracilibacteria;o__Absconditabacteriales_(SR1);f__Absconditabacteriales_(SR1);g__Absconditabacteriales_(SR1)	0.015	0.017	0.022	0.034	0.09619
Bacteria;p__Firmicutes;c__Clostridia;o__Lachnospirales;f__Lachnospiraceae;g__Blautia	0.127	0.031	0.141	0.047	0.09798
Bacteria;p__Actinobacteriota;c__Coriobacteriia;o__Coriobacteriales;f__Atopobiaceae;__	0.054	0.039	0.085	0.093	0.09798
Bacteria;p__Actinobacteriota;c__Actinobacteria;o__Bifidobacteriales;f__Bifidobacteriaceae;g__uncultured	0.054	0.081	0.086	0.176	0.09798

*TP-U/TP-PRU vs NPN-U-PRU/NPN-PRU (Soybean meal replacement by NPN) using a Friedman test.

Table S2. Median and IQR of ruminal archaea and bacterial abundance at genera level influenced by low and high post-ruminal urea (PRU) in the supplement of grazing Nellore steers.

Genera	NPN-PRU	IQR	NPN-U-PRU	IQR	p-value*
Archaea;p__Thermoplasmatota;c__Thermoplasmata;o__Methanomassiliicoccales;f__Methanomethylophilaceae;g__uncultured	0.067	0.052	0.165	0.130	0.0117
Bacteria;p__Firmicutes;c__Clostridia;o__Clostridia;f__Hungateiclostridiaceae;g__Saccharofermentans	1.931	0.527	2.307	0.504	0.0117
Bacteria;p__Bacteroidota;c__Bacteroidia;o__Bacteroidales;f__Bacteroidetes_BD2-2;g__Bacteroidetes_BD2-2	0.128	0.036	0.164	0.027	0.0173
Bacteria;p__Firmicutes;c__Clostridia;o__Oscillospirales;f__Oscillospiraceae;g__Papillibacter	0.024	0.027	0.000	0.000	0.0277
Bacteria;p__Firmicutes;c__Clostridia;o__Peptostreptococcales-Tissierellales;f__Anaerovoracaceae;g__Mogibacterium	0.304	0.040	0.242	0.054	0.0280
Bacteria;p__Actinobacteriota;c__Coriobacteriia;o__Coriobacteriales;f__Atopobiaceae;__	0.077	0.041	0.040	0.028	0.0357
Bacteria;p__Myxococcota;c__Myxococcia;o__Myxococcales;f__Myxococcaceae;__	0.000	0.000	0.003	0.007	0.0412
Bacteria;p__Proteobacteria;c__Gammaproteobacteria;o__Aeromonadales;f__Succinivibrionaceae;g__Succinivibrionaceae_UCG-002	0.021	0.008	0.059	0.084	0.0499
Bacteria;p__Firmicutes;c__Clostridia;o__Lachnospirales;f__Lachnospiraceae;g__[Eubacterium]_ruminantium_group	0.698	0.293	0.778	0.141	0.0499
Bacteria;p__Actinobacteriota;c__Actinobacteria;o__Corynebacteriales;f__Corynebacteriaceae;g__Corynebacterium	0.015	0.011	0.003	0.012	0.0499
Bacteria; p__Firmicutes;c__Clostridia;o__Oscillospirales;__;__	0.756	0.223	1.061	0.410	0.0499
Bacteria;p__Firmicutes;c__Bacilli;o__Erysipelotrichales;f__Erysipelotrichaceae;__	0.009	0.010	0.000	0.001	0.0578
Bacteria;p__Firmicutes;c__Clostridia;o__Eubacteriales;f__Eubacteriaceae;g__Eubacterium	0.000	0.001	0.007	0.019	0.0585
Bacteria;p__Firmicutes;c__Clostridia;o__Peptostreptococcales-Tissierellales;__;__	0.013	0.012	0.005	0.014	0.0630
Bacteria;p__Firmicutes;c__Syntrophomonadia;o__Syntrophomonadales;f__Syntrophomonadaceae;g__Syntrophomonas	0.000	0.000	0.004	0.006	0.0656
Bacteria;p__Firmicutes;c__Clostridia;o__Clostridiales;f__Clostridiaceae;g__Clostridium_sensu_stricto_1	0.007	0.018	0.000	0.000	0.0679
Bacteria;p__Proteobacteria;c__Gammaproteobacteria;o__Aeromonadales;f__Succinivibrionaceae;g__Ruminobacter	0.016	0.003	0.008	0.005	0.0684
Bacteria;p__Firmicutes;c__Clostridia;o__Lachnospirales;f__Lachnospiraceae;g__[Ruminococcus]_gauvreauii_group	0.207	0.192	0.162	0.080	0.0687
Bacteria;p__Proteobacteria;c__Alphaproteobacteria;o__Rickettsiales;f__uncultured;g__uncultured	0.051	0.028	0.082	0.059	0.0687
Bacteria;p__Bacteroidota;c__Bacteroidia;o__Bacteroidales;f__Muribaculaceae;g__Muribaculaceae	0.360	0.089	0.256	0.098	0.0687
Bacteria;p__Firmicutes;c__Clostridia;o__Oscillospirales;f__Oscillospiraceae;g__uncultured	0.151	0.029	0.111	0.016	0.0687
Bacteria;p__Firmicutes;c__Clostridia;o__Christensenellales;f__Christensenellaceae;g__Christensenellaceae_R-7_group	12.031	4.131	10.795	3.352	0.0687
Bacteria;p__Actinobacteriota;c__Coriobacteriia;o__Coriobacteriales;f__Atopobiaceae;g__Olsenella	0.532	0.200	0.458	0.133	0.0929
Bacteria;p__Spirochaetota;c__Spirochaetia;o__Spirochaetales;f__Spirochaetaceae;g__Treponema	0.815	0.515	1.562	0.944	0.0929
Bacteria;p__Firmicutes;c__Clostridia;o__Peptostreptococcales-Tissierellales;f__Anaerovoracaceae;g__Amnipila	0.136	0.014	0.122	0.028	0.0929
Bacteria;p__Firmicutes;c__Clostridia;o__Clostridia;f__Hungateiclostridiaceae;__	0.053	0.029	0.029	0.025	0.0929

* NPN-U-PRU vs NPN-PRU (low and high post-ruminal urea (PRU) level using a paired Wilcoxon signed rank sum test.

Table S3. Median and IQR of ruminal archaea and bacterial abundance at genera level influenced by conventional urea or post-ruminal urea (PRU) in the supplement of grazing Nellore steers.

Genera	TP-PRU	IQR	TP-U	IQR	p-value *
Bacteria;p__Firmicutes;c__Clostridia;o__Peptostreptococcales-Tissierellales;f__Anaerovoracaceae;g__[Eubacterium]_nodatum_group	0.170	0.105	0.307	0.186	0.0117
Bacteria;p__Bacteroidota;c__Bacteroidia;o__Bacteroidales;__;__	1.160	0.074	1.382	0.363	0.0117
Bacteria;p__Firmicutes;c__Clostridia;o__Lachnospirales;f__Lachnospiraceae;g__[Eubacterium]_hallii_group	0.976	0.113	1.197	0.154	0.0172
Bacteria;p__Firmicutes;c__Clostridia;o__Peptostreptococcales-Tissierellales;f__Anaerovoracaceae;g__Mogibacterium	0.289	0.059	0.333	0.046	0.0173
Bacteria;p__Firmicutes;c__Clostridia;o__Oscillospirales;f__Oscillospiraceae;g__Oscillospira	0.015	0.019	0.064	0.046	0.0173
Bacteria;p__Firmicutes;c__Bacilli;o__Erysipelotrichales;f__Erysipelotrichaceae;g__Turicibacter	0.005	0.109	0.000	0.001	0.0273
Bacteria;p__Firmicutes;c__Clostridia;o__Oscillospirales;f__Oscillospiraceae;g__Intestinimonas	0.000	0.000	0.010	0.014	0.0277
Bacteria;p__Desulfobacterota;c__Desulfovibrionia;o__Desulfovibrionales;f__Desulfovibrionaceae;g__Mailhella	0.025	0.012	0.031	0.013	0.0298
Bacteria;p__Actinobacteriota;c__Coriobacteriia;o__Coriobacteriales;f__Atopobiaceae;g__Olsenella	0.506	0.189	0.739	0.211	0.0357
Bacteria;p__Firmicutes;c__Negativicutes;o__Acidaminococcales;f__Acidaminococcaceae;g__Succiniclasticum	1.303	0.180	1.690	0.688	0.0499
Bacteria;p__Firmicutes;c__Clostridia;o__Peptococcales;f__Peptococcaceae;g__uncultured	0.009	0.006	0.000	0.008	0.0630
Bacteria;p__Firmicutes;c__Bacilli;o__Erysipelotrichales;f__Erysipelatoclostridiaceae;g__Erysipelotrichaceae_UCG-002	0.002	0.023	0.000	0.000	0.0679
Bacteria;p__Actinobacteriota;c__Actinobacteria;o__Corynebacteriales;f__Corynebacteriaceae;g__Corynebacterium	0.014	0.016	0.023	0.013	0.0684
Bacteria;p__Firmicutes;c__Clostridia;o__Lachnospirales;f__Lachnospiraceae;g__[Ruminococcus]_gauvreauii_group	0.227	0.196	0.403	0.308	0.0687
Bacteria;p__Bacteroidota;c__Bacteroidia;o__Bacteroidales;f__uncultured;g__uncultured	0.183	0.063	0.265	0.080	0.0687
Bacteria;p__Verrucomicrobiota;c__Lentisphaeria;o__Victivallales;f__vadinBE97;g__vadinBE97	0.009	0.004	0.013	0.003	0.0687
Bacteria;p__Bacteroidota;c__Bacteroidia;o__Bacteroidales;f__Bacteroidales_BS11_gut_group;g__Bacteroidales_BS11_gut_group	0.362	0.211	0.584	0.185	0.0687
Bacteria;p__Firmicutes;c__Clostridia;o__Lachnospirales;f__Lachnospiraceae;g__Stomatobaculum	0.271	0.030	0.305	0.110	0.0687
Bacteria;p__Verrucomicrobiota;c__Kiritimatiellae;o__WCHB1-41;f__WCHB1-41;g__WCHB1-41	0.016	0.021	0.026	0.013	0.0754
Bacteria;p__Firmicutes;c__Bacilli;o__Erysipelotrichales;f__Erysipelotrichaceae;g__Erysipelothrix	0.000	0.000	0.002	0.006	0.0782
Bacteria;p__Firmicutes;c__Clostridia;o__Oscillospirales;f__Ruminococcaceae;g__CAG-352	0.000	0.002	0.006	0.014	0.0796
Bacteria;p__Elusimicrobiota;c__Endomicrobia;o__Endomicrobiales;f__Endomicrobiaceae;g__Endomicrobium	0.010	0.015	0.020	0.013	0.0910
Bacteria;p__Firmicutes;c__Clostridia;o__Peptostreptococcales-Tissierellales;f__Anaerovoracaceae;g__Amnipila	0.104	0.026	0.141	0.055	0.0929
Bacteria;p__Firmicutes;c__Clostridia;o__Peptostreptococcales-Tissierellales;f__Anaerovoracaceae;g__Anaerovorax	0.363	0.045	0.381	0.088	0.0929
Bacteria;p__Firmicutes;c__Clostridia;o__Oscillospirales;f__[Eubacterium]_coprostanoligenes_group;g__[Eubacterium]_coprostanoligenes_group	1.180	0.404	1.344	0.292	0.0929
Bacteria;p__Bacteroidota;c__Bacteroidia;o__Bacteroidales;f__Bacteroidales_RF16_group;g__Bacteroidales_RF16_group	0.450	0.105	0.482	0.046	0.0929

*TP-U vs TP-PRU (conventional urea vs PRU) using a paired Wilcoxon signed rank sum test
